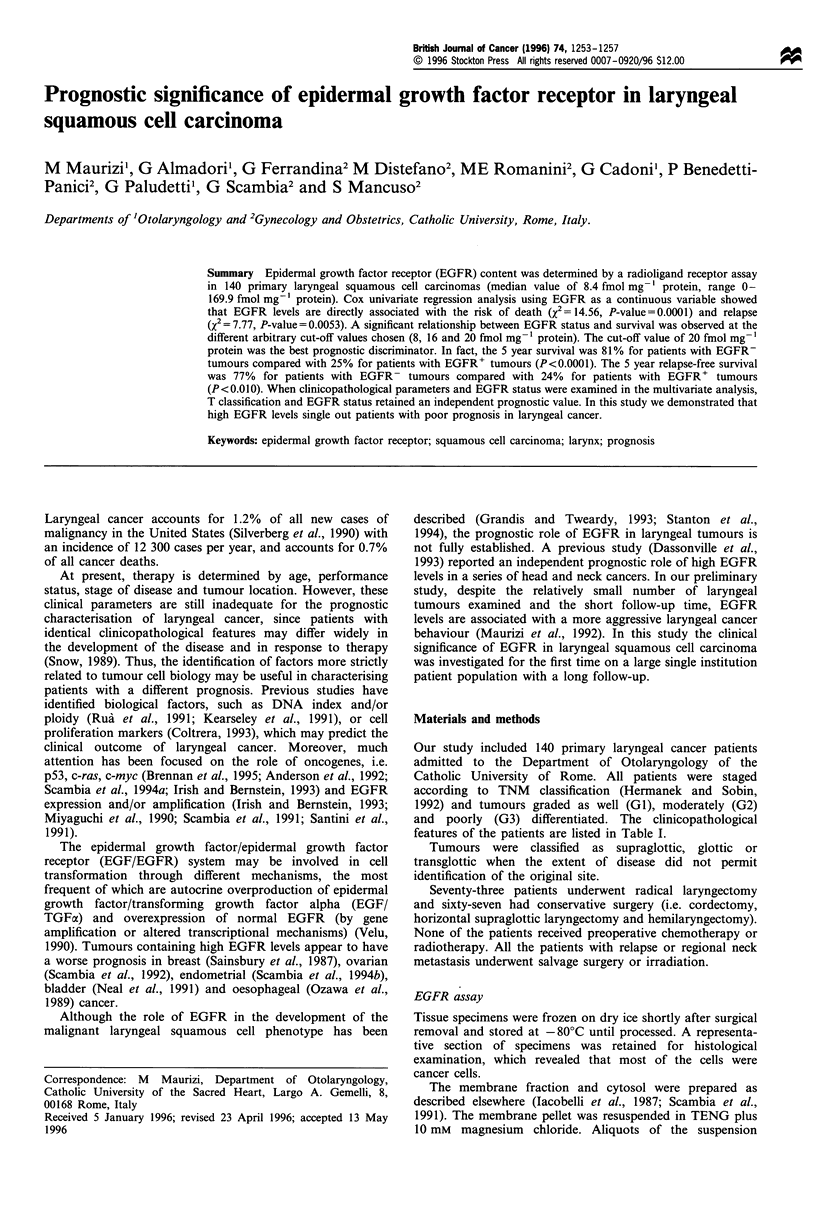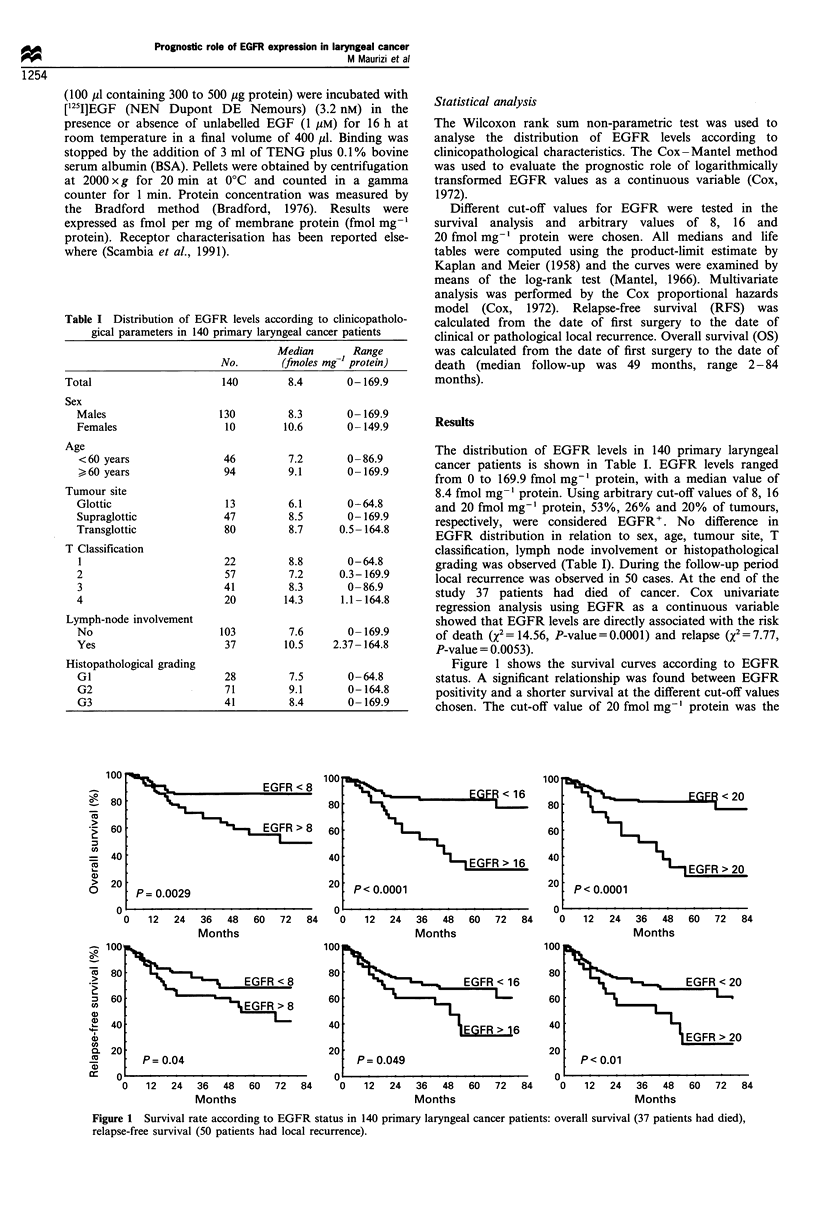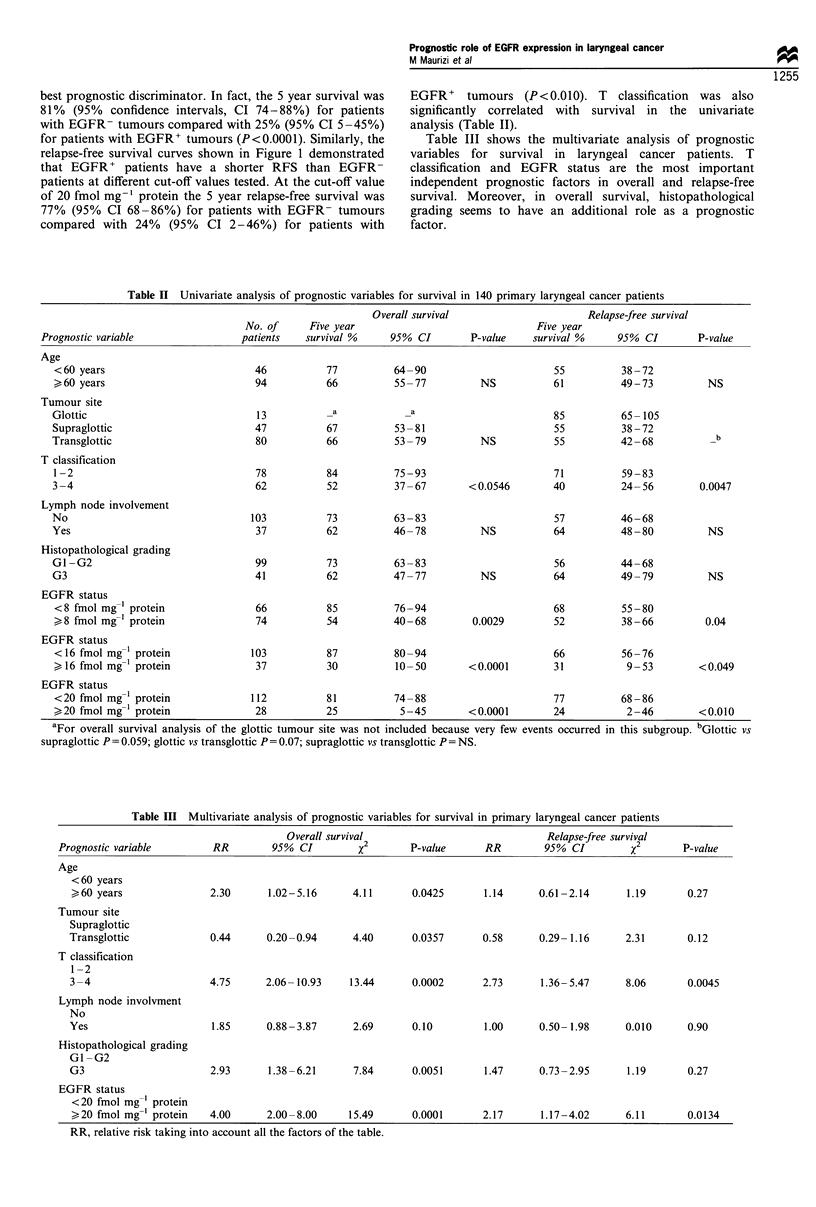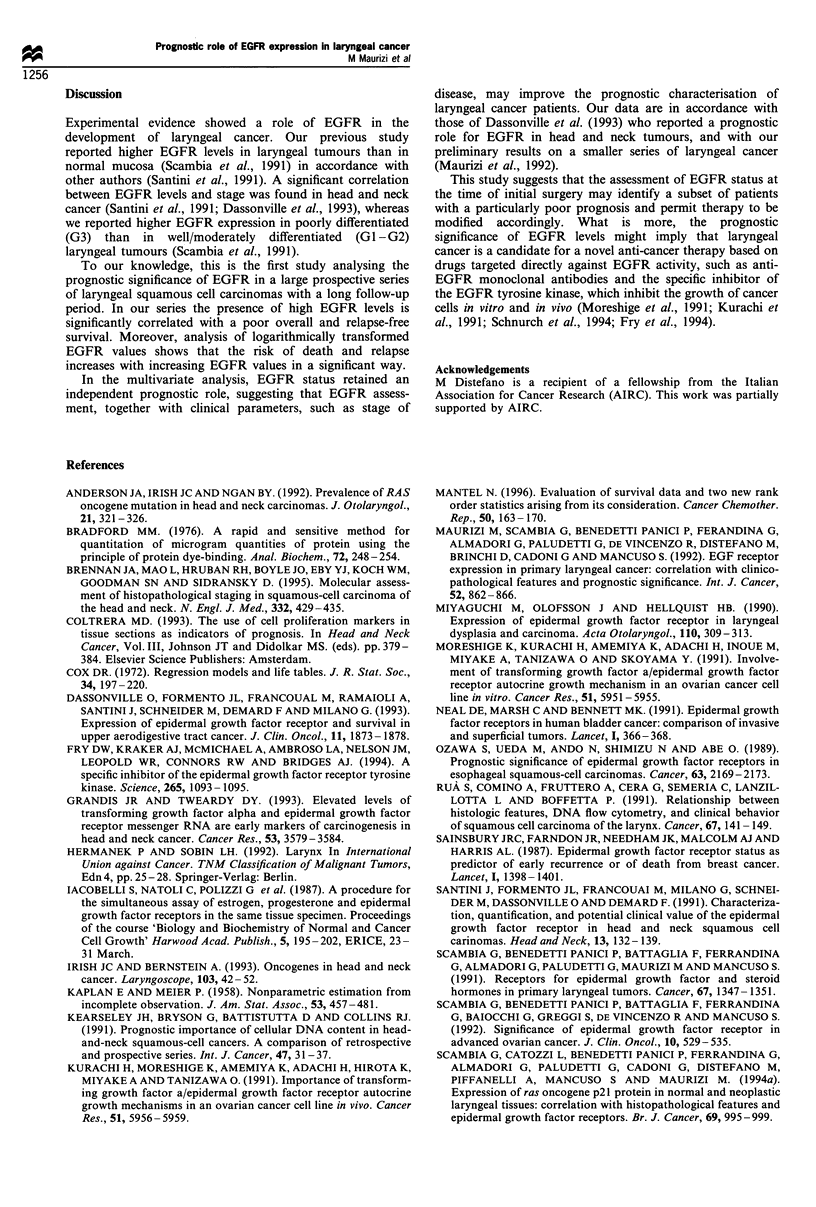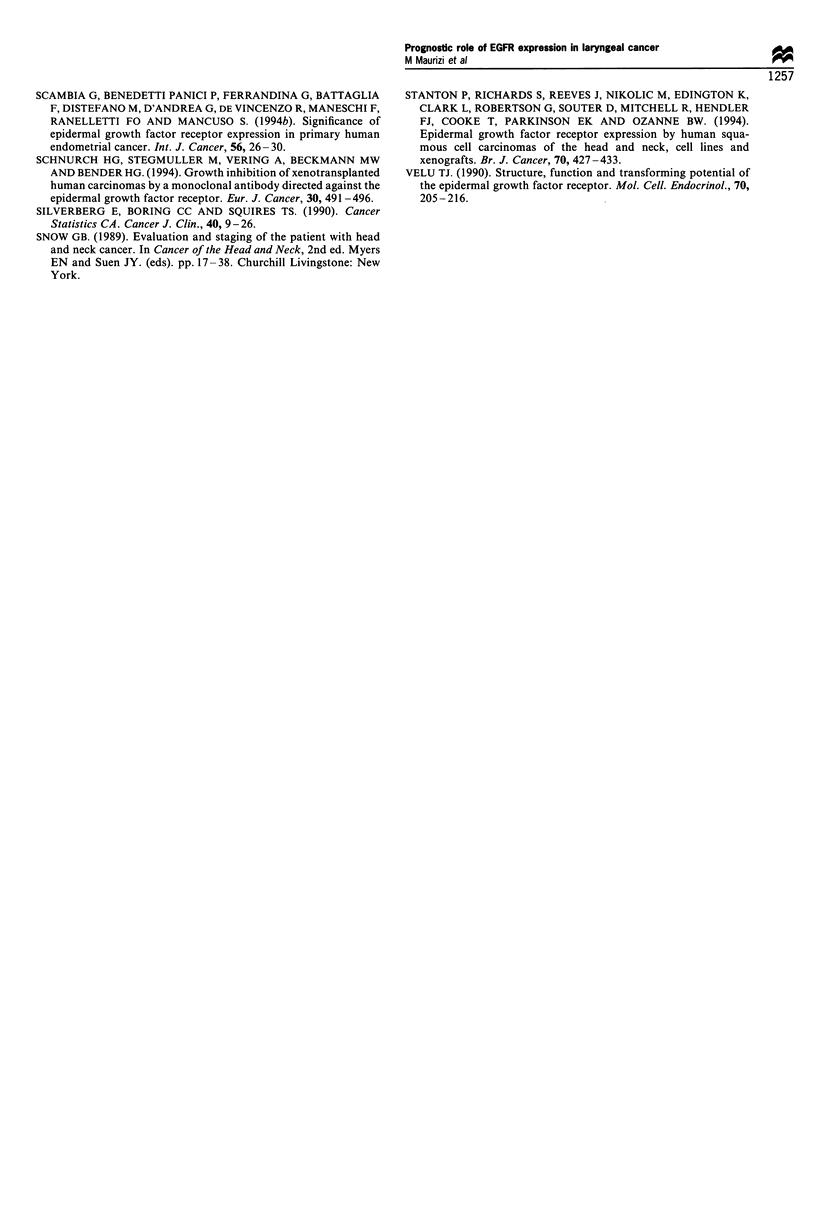# Prognostic significance of epidermal growth factor receptor in laryngeal squamous cell carcinoma.

**DOI:** 10.1038/bjc.1996.525

**Published:** 1996-10

**Authors:** M. Maurizi, G. Almadori, G. Ferrandina, M. Distefano, M. E. Romanini, G. Cadoni, P. Benedetti-Panici, G. Paludetti, G. Scambia, S. Mancuso

**Affiliations:** Department of Otolaryngology, Catholic University, Rome, Italy.

## Abstract

Epidermal growth factor receptor (EGFR) content was determined by a radioligand receptor assay in 140 primary laryngeal squamous cell carcinomas (median value of 8.4 fmol mg-1 protein, range 0-169.9 fmol mg-1 protein). Cox univariate regression analysis using EGFR as a continuous variable showed that EGFR levels are directly associated with the risk of death (chi 2 = 14.56, P-value = 0.0001) and relapse (chi 2 = 7.77, P-value = 0.0053). A significant relationship between EGFR status and survival was observed at the different arbitrary cut-off values chosen (8, 16 and 20 fmol mg-1 protein). The cut-off value of 20 fmol mg-1 protein was the best prognostic discriminator. In fact, the 5 year survival was 81% for patients with EGFR- tumours compared with 25% for patients with EGFR+ tumours (P < 0.0001). The 5 year relapse-free survival was 77% for patients with EGFR- tumours compared with 24% for patients with EGFR+ tumours (P < 0.010). When clinicopathological parameters and EGFR status were examined in the multivariate analysis, T classification and EGFR status retained an independent prognostic value. In this study we demonstrated that high EGFR levels single out patients with poor prognosis in laryngeal cancer.


British Journal of Cancer (1996) 74, 1253-1257
© 1996 Stockton Press All rights reserved 0007-0920/96 $12.00

### Prognostic significance of epidermal growth factor receptor in laryngeal squamous cell carcinoma

M Maurizi[1], G Almadori[1], G Ferrandina[2] M Distefano[2], ME Romanini[2], G Cadoni[1], P Benedetti-Panici[2], G Paludetti[1], G Scambia[2] and S Mancuso[2]

*Departments of [1]Otolaryngology and [2]Gynecology and Obstetrics, Catholic University, Rome, Italy.*

**Summary** Epidermal growth factor receptor (EGFR) content was determined by a radioligand receptor assay in 140 primary laryngeal squamous cell carcinomas (median value of 8.4 fmol $mg^{-1}$ protein, range 0–169.9 fmol $mg^{-1}$ protein). Cox univariate regression analysis using EGFR as a continuous variable showed that EGFR levels are directly associated with the risk of death ($\chi^2=14.56$, $P$-value=0.0001) and relapse ($\chi^2=7.77$, $P$-value=0.0053). A significant relationship between EGFR status and survival was observed at the different arbitrary cut-off values chosen (8, 16 and 20 fmol $mg^{-1}$ protein). The cut-off value of 20 fmol $mg^{-1}$ protein was the best prognostic discriminator. In fact, the 5 year survival was 81% for patients with $EGFR^-$ tumours compared with 25% for patients with $EGFR^+$ tumours ($P<0.0001$). The 5 year relapse-free survival was 77% for patients with $EGFR^-$ tumours compared with 24% for patients with $EGFR^+$ tumours ($P<0.010$). When clinicopathological parameters and EGFR status were examined in the multivariate analysis, T classification and EGFR status retained an independent prognostic value. In this study we demonstrated that high EGFR levels single out patients with poor prognosis in laryngeal cancer.

**Keywords:** epidermal growth factor receptor; squamous cell carcinoma; larynx; prognosis

Laryngeal cancer accounts for 1.2% of all new cases of malignancy in the United States (Silverberg *et al.*, 1990) with an incidence of 12 300 cases per year, and accounts for 0.7% of all cancer deaths.

At present, therapy is determined by age, performance status, stage of disease and tumour location. However, these clinical parameters are still inadequate for the prognostic characterisation of laryngeal cancer, since patients with identical clinicopathological features may differ widely in the development of the disease and in response to therapy (Snow, 1989). Thus, the identification of factors more strictly related to tumour cell biology may be useful in characterising patients with a different prognosis. Previous studies have identified biological factors, such as DNA index and/or ploidy (Ruà *et al.*, 1991; Kearseley *et al.*, 1991), or cell proliferation markers (Coltrera, 1993), which may predict the clinical outcome of laryngeal cancer. Moreover, much attention has been focused on the role of oncogenes, i.e. p53, c-*ras*, c-*myc* (Brennan *et al.*, 1995; Anderson *et al.*, 1992; Scambia *et al.*, 1994*a*; Irish and Bernstein, 1993) and EGFR expression and/or amplification (Irish and Bernstein, 1993; Miyaguchi *et al.*, 1990; Scambia *et al.*, 1991; Santini *et al.*, 1991).

The epidermal growth factor/epidermal growth factor receptor (EGF/EGFR) system may be involved in cell transformation through different mechanisms, the most frequent of which are autocrine overproduction of epidermal growth factor/transforming growth factor alpha (EGF/TGFα) and overexpression of normal EGFR (by gene amplification or altered transcriptional mechanisms) (Velu, 1990). Tumours containing high EGFR levels appear to have a worse prognosis in breast (Sainsbury *et al.*, 1987), ovarian (Scambia *et al.*, 1992), endometrial (Scambia *et al.*, 1994*b*), bladder (Neal *et al.*, 1991) and oesophageal (Ozawa *et al.*, 1989) cancer.

Although the role of EGFR in the development of the malignant laryngeal squamous cell phenotype has been described (Grandis and Tweardy, 1993; Stanton *et al.*, 1994), the prognostic role of EGFR in laryngeal tumours is not fully established. A previous study (Dassonville *et al.*, 1993) reported an independent prognostic role of high EGFR levels in a series of head and neck cancers. In our preliminary study, despite the relatively small number of laryngeal tumours examined and the short follow-up time, EGFR levels are associated with a more aggressive laryngeal cancer behaviour (Maurizi *et al.*, 1992). In this study the clinical significance of EGFR in laryngeal squamous cell carcinoma was investigated for the first time on a large single institution patient population with a long follow-up.

### Materials and methods

Our study included 140 primary laryngeal cancer patients admitted to the Department of Otolaryngology of the Catholic University of Rome. All patients were staged according to TNM classification (Hermanek and Sobin, 1992) and tumours graded as well (G1), moderately (G2) and poorly (G3) differentiated. The clinicopathological features of the patients are listed in Table I.

Tumours were classified as supraglottic, glottic or transglottic when the extent of disease did not permit identification of the original site.

Seventy-three patients underwent radical laryngectomy and sixty-seven had conservative surgery (i.e. cordectomy, horizontal supraglottic laryngectomy and hemilaryngectomy). None of the patients received preoperative chemotherapy or radiotherapy. All the patients with relapse or regional neck metastasis underwent salvage surgery or irradiation.

### *EGFR assay*

Tissue specimens were frozen on dry ice shortly after surgical removal and stored at −80°C until processed. A representative section of specimens was retained for histological examination, which revealed that most of the cells were cancer cells.

The membrane fraction and cytosol were prepared as described elsewhere (Iacobelli *et al.*, 1987; Scambia *et al.*, 1991). The membrane pellet was resuspended in TENG plus 10 mM magnesium chloride. Aliquots of the suspension

Correspondence: M Maurizi, Department of Otolaryngology, Catholic University of the Sacred Heart, Largo A. Gemelli, 8, 00168 Rome, Italy
Received 5 January 1996; revised 23 April 1996; accepted 13 May 1996

(100 μl containing 300 to 500 μg protein) were incubated with [$^{125}$I]EGF (NEN Dupont DE Nemours) (3.2 nM) in the presence or absence of unlabelled EGF (1 μM) for 16 h at room temperature in a final volume of 400 μl. Binding was stopped by the addition of 3 ml of TENG plus 0.1% bovine serum albumin (BSA). Pellets were obtained by centrifugation at 2000 × *g* for 20 min at 0°C and counted in a gamma counter for 1 min. Protein concentration was measured by the Bradford method (Bradford, 1976). Results were expressed as fmol per mg of membrane protein (fmol $mg^{-1}$ protein). Receptor characterisation has been reported elsewhere (Scambia *et al.*, 1991).

**Table I** Distribution of EGFR levels according to clinicopathological parameters in 140 primary laryngeal cancer patients

| | *No.* | *Median (fmoles mg$^{-1}$* | *Range protein)* |
|---|---|---|---|
| Total | 140 | 8.4 | 0–169.9 |
| Sex | | | |
| Males | 130 | 8.3 | 0–169.9 |
| Females | 10 | 10.6 | 0–149.9 |
| Age | | | |
| <60 years | 46 | 7.2 | 0–86.9 |
| ⩾60 years | 94 | 9.1 | 0–169.9 |
| Tumour site | | | |
| Glottic | 13 | 6.1 | 0–64.8 |
| Supraglottic | 47 | 8.5 | 0–169.9 |
| Transglottic | 80 | 8.7 | 0.5–164.8 |
| T Classification | | | |
| 1 | 22 | 8.8 | 0–64.8 |
| 2 | 57 | 7.2 | 0.3–169.9 |
| 3 | 41 | 8.3 | 0–86.9 |
| 4 | 20 | 14.3 | 1.1–164.8 |
| Lymph-node involvement | | | |
| No | 103 | 7.6 | 0–169.9 |
| Yes | 37 | 10.5 | 2.37–164.8 |
| Histopathological grading | | | |
| G1 | 28 | 7.5 | 0–64.8 |
| G2 | 71 | 9.1 | 0–164.8 |
| G3 | 41 | 8.4 | 0–169.9 |

*Statistical analysis*

The Wilcoxon rank sum non-parametric test was used to analyse the distribution of EGFR levels according to clinicopathological characteristics. The Cox–Mantel method was used to evaluate the prognostic role of logarithmically transformed EGFR values as a continuous variable (Cox, 1972).

Different cut-off values for EGFR were tested in the survival analysis and arbitrary values of 8, 16 and 20 fmol $mg^{-1}$ protein were chosen. All medians and life tables were computed using the product-limit estimate by Kaplan and Meier (1958) and the curves were examined by means of the log-rank test (Mantel, 1966). Multivariate analysis was performed by the Cox proportional hazards model (Cox, 1972). Relapse-free survival (RFS) was calculated from the date of first surgery to the date of clinical or pathological local recurrence. Overall survival (OS) was calculated from the date of first surgery to the date of death (median follow-up was 49 months, range 2–84 months).

### Results

The distribution of EGFR levels in 140 primary laryngeal cancer patients is shown in Table I. EGFR levels ranged from 0 to 169.9 fmol $mg^{-1}$ protein, with a median value of 8.4 fmol $mg^{-1}$ protein. Using arbitrary cut-off values of 8, 16 and 20 fmol $mg^{-1}$ protein, 53%, 26% and 20% of tumours, respectively, were considered EGFR$^+$. No difference in EGFR distribution in relation to sex, age, tumour site, T classification, lymph node involvement or histopathological grading was observed (Table I). During the follow-up period local recurrence was observed in 50 cases. At the end of the study 37 patients had died of cancer. Cox univariate regression analysis using EGFR as a continuous variable showed that EGFR levels are directly associated with the risk of death ($\chi^2$ = 14.56, *P*-value = 0.0001) and relapse ($\chi^2$ = 7.77, *P*-value = 0.0053).

Figure 1 shows the survival curves according to EGFR status. A significant relationship was found between EGFR positivity and a shorter survival at the different cut-off values chosen. The cut-off value of 20 fmol $mg^{-1}$ protein was the

**Figure 1** Survival rate according to EGFR status in 140 primary laryngeal cancer patients: overall survival (37 patients had died), relapse-free survival (50 patients had local recurrence).

best prognostic discriminator. In fact, the 5 year survival was 81% (95% confidence intervals, CI 74–88%) for patients with $EGFR^-$ tumours compared with 25% (95% CI 5–45%) for patients with $EGFR^+$ tumours ($P<0.0001$). Similarly, the relapse-free survival curves shown in Figure 1 demonstrated that $EGFR^+$ patients have a shorter RFS than $EGFR^-$ patients at different cut-off values tested. At the cut-off value of 20 fmol $mg^{-1}$ protein the 5 year relapse-free survival was 77% (95% CI 68–86%) for patients with $EGFR^-$ tumours compared with 24% (95% CI 2–46%) for patients with $EGFR^+$ tumours ($P<0.010$). T classification was also significantly correlated with survival in the univariate analysis (Table II).

Table III shows the multivariate analysis of prognostic variables for survival in laryngeal cancer patients. T classification and EGFR status are the most important independent prognostic factors in overall and relapse-free survival. Moreover, in overall survival, histopathological grading seems to have an additional role as a prognostic factor.

**Table II** Univariate analysis of prognostic variables for survival in 140 primary laryngeal cancer patients

| | | *Overall survival* | | | *Relapse-free survival* | | |
|---|---|---|---|---|---|---|---|
| *Prognostic variable* | *No. of patients* | *Five year survival %* | *95% CI* | P-*value* | *Five year survival %* | *95% CI* | P-*value* |
| Age | | | | | | | |
| <60 years | 46 | 77 | 64–90 | | 55 | 38–72 | |
| ⩾60 years | 94 | 66 | 55–77 | NS | 61 | 49–73 | NS |
| Tumour site | | | | | | | |
| Glottic | 13 | –[a] | –[a] | | 85 | 65–105 | |
| Supraglottic | 47 | 67 | 53–81 | | 55 | 38–72 | |
| Transglottic | 80 | 66 | 53–79 | NS | 55 | 42–68 | –[b] |
| T classification | | | | | | | |
| 1–2 | 78 | 84 | 75–93 | | 71 | 59–83 | |
| 3–4 | 62 | 52 | 37–67 | <0.0546 | 40 | 24–56 | 0.0047 |
| Lymph node involvement | | | | | | | |
| No | 103 | 73 | 63–83 | | 57 | 46–68 | |
| Yes | 37 | 62 | 46–78 | NS | 64 | 48–80 | NS |
| Histopathological grading | | | | | | | |
| G1–G2 | 99 | 73 | 63–83 | | 56 | 44–68 | |
| G3 | 41 | 62 | 47–77 | NS | 64 | 49–79 | NS |
| EGFR status | | | | | | | |
| <8 fmol $mg^{-1}$ protein | 66 | 85 | 76–94 | | 68 | 55–80 | |
| ⩾8 fmol $mg^{-1}$ protein | 74 | 54 | 40–68 | 0.0029 | 52 | 38–66 | 0.04 |
| EGFR status | | | | | | | |
| <16 fmol $mg^{-1}$ protein | 103 | 87 | 80–94 | | 66 | 56–76 | |
| ⩾16 fmol $mg^{-1}$ protein | 37 | 30 | 10–50 | <0.0001 | 31 | 9–53 | <0.049 |
| EGFR status | | | | | | | |
| <20 fmol $mg^{-1}$ protein | 112 | 81 | 74–88 | | 77 | 68–86 | |
| ⩾20 fmol $mg^{-1}$ protein | 28 | 25 | 5–45 | <0.0001 | 24 | 2–46 | <0.010 |

[a]For overall survival analysis of the glottic tumour site was not included because very few events occurred in this subgroup. [b]Glottic *vs* supraglottic $P=0.059$; glottic *vs* transglottic $P=0.07$; supraglottic *vs* transglottic $P=$ NS.

**Table III** Multivariate analysis of prognostic variables for survival in primary laryngeal cancer patients

| | *Overall survival* | | | | *Relapse-free survival* | | | |
|---|---|---|---|---|---|---|---|---|
| *Prognostic variable* | *RR* | *95% CI* | $\chi^2$ | P-*value* | *RR* | *95% CI* | $\chi^2$ | P-*value* |
| Age | | | | | | | | |
| <60 years | | | | | | | | |
| ⩾60 years | 2.30 | 1.02–5.16 | 4.11 | 0.0425 | 1.14 | 0.61–2.14 | 1.19 | 0.27 |
| Tumour site | | | | | | | | |
| Supraglottic | | | | | | | | |
| Transglottic | 0.44 | 0.20–0.94 | 4.40 | 0.0357 | 0.58 | 0.29–1.16 | 2.31 | 0.12 |
| T classification | | | | | | | | |
| 1–2 | | | | | | | | |
| 3–4 | 4.75 | 2.06–10.93 | 13.44 | 0.0002 | 2.73 | 1.36–5.47 | 8.06 | 0.0045 |
| Lymph node involvment | | | | | | | | |
| No | | | | | | | | |
| Yes | 1.85 | 0.88–3.87 | 2.69 | 0.10 | 1.00 | 0.50–1.98 | 0.010 | 0.90 |
| Histopathological grading | | | | | | | | |
| G1–G2 | | | | | | | | |
| G3 | 2.93 | 1.38–6.21 | 7.84 | 0.0051 | 1.47 | 0.73–2.95 | 1.19 | 0.27 |
| EGFR status | | | | | | | | |
| <20 fmol $mg^{-1}$ protein | | | | | | | | |
| ⩾20 fmol $mg^{-1}$ protein | 4.00 | 2.00–8.00 | 15.49 | 0.0001 | 2.17 | 1.17–4.02 | 6.11 | 0.0134 |

RR, relative risk taking into account all the factors of the table.

### Discussion

Experimental evidence showed a role of EGFR in the development of laryngeal cancer. Our previous study reported higher EGFR levels in laryngeal tumours than in normal mucosa (Scambia *et al.*, 1991) in accordance with other authors (Santini *et al.*, 1991). A significant correlation between EGFR levels and stage was found in head and neck cancer (Santini *et al.*, 1991; Dassonville *et al.*, 1993), whereas we reported higher EGFR expression in poorly differentiated (G3) than in well/moderately differentiated (G1-G2) laryngeal tumours (Scambia *et al.*, 1991).

To our knowledge, this is the first study analysing the prognostic significance of EGFR in a large prospective series of laryngeal squamous cell carcinomas with a long follow-up period. In our series the presence of high EGFR levels is significantly correlated with a poor overall and relapse-free survival. Moreover, analysis of logarithmically transformed EGFR values shows that the risk of death and relapse increases with increasing EGFR values in a significant way.

In the multivariate analysis, EGFR status retained an independent prognostic role, suggesting that EGFR assessment, together with clinical parameters, such as stage of disease, may improve the prognostic characterisation of laryngeal cancer patients. Our data are in accordance with those of Dassonville *et al.* (1993) who reported a prognostic role for EGFR in head and neck tumours, and with our preliminary results on a smaller series of laryngeal cancer (Maurizi *et al.*, 1992).

This study suggests that the assessment of EGFR status at the time of initial surgery may identify a subset of patients with a particularly poor prognosis and permit therapy to be modified accordingly. What is more, the prognostic significance of EGFR levels might imply that laryngeal cancer is a candidate for a novel anti-cancer therapy based on drugs targeted directly against EGFR activity, such as anti-EGFR monoclonal antibodies and the specific inhibitor of the EGFR tyrosine kinase, which inhibit the growth of cancer cells *in vitro* and *in vivo* (Moreshige *et al.*, 1991; Kurachi *et al.*, 1991; Schnurch *et al.*, 1994; Fry *et al.*, 1994).

**Acknowledgements**
M Distefano is a recipient of a fellowship from the Italian Association for Cancer Research (AIRC). This work was partially supported by AIRC.

### References

ANDERSON JA, IRISH JC AND NGAN BY. (1992). Prevalence of *RAS* oncogene mutation in head and neck carcinomas. *J. Otolaryngol.*, **21**, 321-326.

BRADFORD MM. (1976). A rapid and sensitive method for quantitation of microgram quantities of protein using the principle of protein dye-binding. *Anal. Biochem.*, **72**, 248-254.

BRENNAN JA, MAO L, HRUBAN RH, BOYLE JO, EBY YJ, KOCH WM, GOODMAN SN AND SIDRANSKY D. (1995). Molecular assessment of histopathological staging in squamous-cell carcinoma of the head and neck. *N. Engl. J. Med.*, **332**, 429-435.

COLTRERA MD. (1993). The use of cell proliferation markers in tissue sections as indicators of prognosis. In *Head and Neck Cancer*, Vol. III, Johnson JT and Didolkar MS. (eds). pp. 379-384. Elsevier Science Publishers: Amsterdam.

COX DR. (1972). Regression models and life tables. *J. R. Stat. Soc.*, **34**, 197-220.

DASSONVILLE O, FORMENTO JL, FRANCOUAL M, RAMAIOLI A, SANTINI J, SCHNEIDER M, DEMARD F AND MILANO G. (1993). Expression of epidermal growth factor receptor and survival in upper aerodigestive tract cancer. *J. Clin. Oncol.*, **11**, 1873-1878.

FRY DW, KRAKER AJ, MCMICHAEL A, AMBROSO LA, NELSON JM, LEOPOLD WR, CONNORS RW AND BRIDGES AJ. (1994). A specific inhibitor of the epidermal growth factor receptor tyrosine kinase. *Science*, **265**, 1093-1095.

GRANDIS JR AND TWEARDY DY. (1993). Elevated levels of transforming growth factor alpha and epidermal growth factor receptor messenger RNA are early markers of carcinogenesis in head and neck cancer. *Cancer Res.*, **53**, 3579-3584.

HERMANEK P AND SOBIN LH. (1992). Larynx In *International Union against Cancer. TNM Classification of Malignant Tumors*, Edn 4, pp. 25-28. Springer-Verlag: Berlin.

IACOBELLI S, NATOLI C, POLIZZI G *et al.* (1987). A procedure for the simultaneous assay of estrogen, progesterone and epidermal growth factor receptors in the same tissue specimen. Proceedings of the course 'Biology and Biochemistry of Normal and Cancer Cell Growth' *Harwood Acad. Publish.*, **5**, 195-202, ERICE, 23-31 March.

IRISH JC AND BERNSTEIN A. (1993). Oncogenes in head and neck cancer. *Laryngoscope*, **103**, 42-52.

KAPLAN E AND MEIER P. (1958). Nonparametric estimation from incomplete observation. *J. Am. Stat. Assoc.*, **53**, 457-481.

KEARSELEY JH, BRYSON G, BATTISTUTTA D AND COLLINS RJ. (1991). Prognostic importance of cellular DNA content in head-and-neck squamous-cell cancers. A comparison of retrospective and prospective series. *Int. J. Cancer*, **47**, 31-37.

KURACHI H, MORESHIGE K, AMEMIYA K, ADACHI H, HIROTA K, MIYAKE A AND TANIZAWA O. (1991). Importance of transforming growth factor a/epidermal growth factor receptor autocrine growth mechanisms in an ovarian cancer cell line *in vivo*. *Cancer Res.*, **51**, 5956-5959.

MANTEL N. (1996). Evaluation of survival data and two new rank order statistics arising from its consideration. *Cancer Chemother. Rep.*, **50**, 163-170.

MAURIZI M, SCAMBIA G, BENEDETTI PANICI P, FERANDINA G, ALMADORI G, PALUDETTI G, DE VINCENZO R, DISTEFANO M, BRINCHI D, CADONI G AND MANCUSO S. (1992). EGF receptor expression in primary laryngeal cancer: correlation with clinico-pathological features and prognostic significance. *Int. J. Cancer*, **52**, 862-866.

MIYAGUCHI M, OLOFSSON J AND HELLQUIST HB. (1990). Expression of epidermal growth factor receptor in laryngeal dysplasia and carcinoma. *Acta Otolaryngol.*, **110**, 309-313.

MORESHIGE K, KURACHI H, AMEMIYA K, ADACHI H, INOUE M, MIYAKE A, TANIZAWA O AND SKOYAMA Y. (1991). Involvement of transforming growth factor a/epidermal growth factor receptor autocrine growth mechanism in an ovarian cancer cell line *in vitro*. *Cancer Res.*, **51**, 5951-5955.

NEAL DE, MARSH C AND BENNETT MK. (1991). Epidermal growth factor receptors in human bladder cancer: comparison of invasive and superficial tumors. *Lancet*, **I**, 366-368.

OZAWA S, UEDA M, ANDO N, SHIMIZU N AND ABE O. (1989). Prognostic significance of epidermal growth factor receptors in esophageal squamous-cell carcinomas. *Cancer*, **63**, 2169-2173.

RUÀ S, COMINO A, FRUTTERO A, CERA G, SEMERIA C, LANZILLOTTA L AND BOFFETTA P. (1991). Relationship between histologic features, DNA flow cytometry, and clinical behavior of squamous cell carcinoma of the larynx. *Cancer*, **67**, 141-149.

SAINSBURY JRC, FARNDON JR, NEEDHAM JK, MALCOLM AJ AND HARRIS AL. (1987). Epidermal growth factor receptor status as predictor of early recurrence or of death from breast cancer. *Lancet*, **I**, 1398-1401.

SANTINI J, FORMENTO JL, FRANCOUAI M, MILANO G, SCHNEIDER M, DASSONVILLE O AND DEMARD F. (1991). Characterization, quantification, and potential clinical value of the epidermal growth factor receptor in head and neck squamous cell carinomas. *Head and Neck*, **13**, 132-139.

SCAMBIA G, BENEDETTI PANICI P, BATTAGLIA F, FERRANDINA G, ALMADORI G, PALUDETTI G, MAURIZI M AND MANCUSO S. (1991). Receptors for epidermal growth factor and steroid hormones in primary laryngeal tumors. *Cancer*, **67**, 1347-1351.

SCAMBIA G, BENEDETTI PANICI P, BATTAGLIA F, FERRANDINA G, BAIOCCHI G, GREGGI S, DE VINCENZO R AND MANCUSO S. (1992). Significance of epidermal growth factor receptor in advanced ovarian cancer. *J. Clin. Oncol.*, **10**, 529-535.

SCAMBIA G, CATOZZI L, BENEDETTI PANICI P, FERRANDINA G, ALMADORI G, PALUDETTI G, CADONI G, DISTEFANO M, PIFFANELLI A, MANCUSO S AND MAURIZI M. (1994*a*). Expression of *ras* oncogene p21 protein in normal and neoplastic laryngeal tissues: correlation with histopathological features and epidermal growth factor receptors. *Br. J. Cancer*, **69**, 995-999.

SCAMBIA G, BENEDETTI PANICI P, FERRANDINA G, BATTAGLIA F, DISTEFANO M, D'ANDREA G, DE VINCENZO R, MANESCHI F, RANELLETTI FO AND MANCUSO S. (1994*b*). Significance of epidermal growth factor receptor expression in primary human endometrial cancer. *Int. J. Cancer*, **56,** 26–30.

SCHNURCH HG, STEGMULLER M, VERING A, BECKMANN MW AND BENDER HG. (1994). Growth inhibition of xenotransplanted human carcinomas by a monoclonal antibody directed against the epidermal growth factor receptor. *Eur. J. Cancer*, **30,** 491–496.

SILVERBERG E, BORING CC AND SQUIRES TS. (1990). *Cancer Statistics CA. Cancer J. Clin.*, **40,** 9–26.

SNOW GB. (1989). Evaluation and staging of the patient with head and neck cancer. In *Cancer of the Head and Neck*, 2nd ed. Myers EN and Suen JY. (eds). pp. 17–38. Churchill Livingstone: New York.

STANTON P, RICHARDS S, REEVES J, NIKOLIC M, EDINGTON K, CLARK L, ROBERTSON G, SOUTER D, MITCHELL R, HENDLER FJ, COOKE T, PARKINSON EK AND OZANNE BW. (1994). Epidermal growth factor receptor expression by human squamous cell carcinomas of the head and neck, cell lines and xenografts. *Br. J. Cancer*, **70,** 427–433.

VELU TJ. (1990). Structure, function and transforming potential of the epidermal growth factor receptor. *Mol. Cell. Endocrinol.*, **70,** 205–216.